# Evaluation of Pharmaceutical Products of St. John’s Wort Efficacy Added on Tricyclic Antidepressants in treating Major Depressive Disorder: A Double Blind Randomized Control Trial

**Published:** 2012-08-25

**Authors:** Sirus Pakseresht, Hatam Boustani, Mohammad Ebrahim Azemi, Jale Nilsaz, Reza Babapour, Mohammad Reza Haghdust

**Affiliations:** 1 Department of Psychiatry, Jundishapur University of Medical Sciences, Ahvaz, IR Iran; 2 Department of Pharmacognosy, School of Pharmacy, Jundishapur University of Medical Sciences, Ahvaz, IR Iran; 3 Medical Herbs and Natural Products Research Center, Jundishapur University of Medical Sciences, Ahvaz, IR Iran; 4 School of Medicine, Jundishapur University of Medical Sciences, Ahvaz, IR Iran; 5 Golestan Hospital, Jundishapur University of Medical Sciences, Ahvaz, IR Iran

**Keywords:** Depressive Disorder, Major, Antidepressive Agents, Tricyclic

## Abstract

**Background:**

Major depressive disorder (MDD) is the most common disorder in our community. Hypericum perforatumm, is a herb with a long application history in treating depression. A controlled study to evaluate the effects of this herbal medicine in Iran did not exist.

**Objectives:**

This study aimed to assess the effect of Hypericum perforatom (perforan), in combination with tricyclic antidepressants in MDD treatment.

**Materials and Methods:**

The present study was a placebo-controlled double blind randomized clinical trial including 40 patients with major depressive disorder who referred to Psychiatric Clinic and Golestan Hospital in 2011. Patients were randomly distributed in 4 blocks with 10 patients in each. 5 out of 10 patients took medicine (group I) and the others received placebo (group II).

**Results:**

After six weeks, both groups indicated some improvement, there was also a significant degree of recovery in the perforan group (P = 0.04).

**Conclusions:**

The results of the current study suggested that combinations of St. John’s Wort and tricyclic antidepressants compared to tricyclic antidepressants alone had significant effect on mild to moderate depression improvement. According to the numerous side effects of antidepressants, their continuous use was not well tolerated. There is a strong global tendency to use herbal medicines. Hypericum perforatom (St. John’s Wort) is one of the best types of unapproved MDD treatments. Also, the most common reason to discontinue antidepressant treatments is their sexual side effects. None of the patients in the two groups had complained about sexual side effects which was consistent with other studies and it was an advantage of using this herbal medicine. Perforan group had improved in quality of sleep and the increase of energy. This improvement had not been reported in previous studies. Sleep problems are common complications of specific serotonin reuptake inhibitors (SSRIs).

## 1. Background

Depression is the most common psychiatric disorder in the community and other medical disorders (1). One-year prevalence and lifetime major depression, respectively, are estimated about 2 .7% and 19.4% (2). The annual incidence of major depression is 1.59% (women 1.89% and men 1. 1% (3). Javidi showed that in people older than 15 years of Marvdasht villages 7. 8% of people were trapped in a kind of depressive disorder (4). In recent study, the prevalence of major depression has been mentioned 6.24%. Nurbala etal; reported 4. 4 %for major depression in Tehran (5).

The current treatment for depression is medication. The use of specific serotonin reuptake inhibitors (SSRI) in the last 20 years has followed less side effects ,and more acceptance ,by the patients, than the older tricyclic (TCA) (6, 7). However, according to numerous side effects of antidepressants, their continuous use is not well tolerated. there is a strong global tendency to use herbal medicines (8). The number of patients asking their doctors whether they can benefit from natural treatments is increasing and many of the patients see herbalists. One of the best types of unapproved MDD treatments is St. John’s Wort extract herbal medicine (Hypericum perforatom) (9). St. John’s Wort is a herbal medication obtained from the flowering tops of the perennial plant Hypericum perforatom that contains various compounds such as flavonoids and xanthones, anthracene derivatives, caffeic acid derivatives, tannins, and volatile oils (10). Mechanism of Hypericum perforatom action is not well known. Hypericin, one of the main active components in Hypericum perforatom decreases serotonin receptor density, inhibits production of interleukine 6,1β by monocyte, resulting in a decrease in releasing corticotrophin hormone thus dumpening production of cortisol. Hypericin may also inhibit reuptake of serotonin, norepinephrine and dopamine and may thus result in reduced expression of beta adrenoreceptores and increased density of serotonin receptors .Hypericin may also have affinity to GABA receptors (11).

Hypericum perforatom is a mono amino oxidase inhibitor and can stimulate GABA receptors, and is attached seroton in 5HT1 receptor. In a study including over two hundred non-psychotic depressed patients St. John’s Wort extract was compared versus placebo. Both groups showed improvement but no significant improvement versus placebo (12). In another study which was done on two groups of depressed patients with the control placebo, St. John’s Wort improved depression significantly compared to placebo (13). Linde et al. performed a meta-analysis on 23 randomized clinical trials of Hypericum extracts with that of patients who suffered from mild or moderate depressive disorder. The results revealed a significantly higher responder under Hypericum in comparison to placebo and equal responder rate compared to the other antidepressants (14). Systemic review and meta-analysis of 30 RCTs indicated that certain St. John’s Wort extracts were more effective than placebo and as effective as certain conventional antidepressants (15). In the treatment of moderate to severe major depression in double blind control study, Hypericum extract was at least as effective as paroxetine and was better tolerated, however, this study could not demonstrate non-inferiority of the herbal extract (16).

The most common adverse reaction of Hypericum in adults is sun sensitivity. Other side effects include allergic reactions, constipation, light headedness, dry mouth, restlessness, gastrointestinal discomfort, sleep disturbance (10) weakness (17) and headaches (18). Adverse drug reaction is very low and uncommon. In one study, about 2.4% of patients mentioned allergic reaction and gastrointestinal complains. Only 1.5% of patients stopped taking the drug, because of side effects (11). Orthostatic hypotension is common with TCAs and related drugs, and is not reported as a significant adverse effect of St. Johns Wort (16). St. John’s Wort decreased action of warfarin, ocp, cyclosporine and theophyline. It’s also recommended that St. John’s Wort not be combined with SSRIs (11). It is likely that there is also drug interference between St. John’s Wort, tryptans (19), tramadol (20), and anticonvulsants (15).

As Hypericum perforatom is now available in the country’s pharmaceutical market without a prescription, lack of control and study on this pharmaceutical product was the researchers` concern. It is known that processing method can alter plant compounds so other countries’ studies could not be used. Also, the most common reason to discontinue antidepressant treatments is sexual side effects of SSRIs which are very common in most patients, but they have not been reported in various studies with hypericum perforatom. Comparing anti depression effects of hypericum perforatom (perforan), and that of the placebo in reducing the symptoms of depression was interesting.

## 2. Objectives

This study aimed to assess the effect of Hypericum perforatom (perforan), in combination with tricyclic antidepressant in MDD treatment.

## 3. Materials and Methods

This study was a double blind randomized clinical trial (RCT) including 40 patients with depression, aged 18-54 years old, who referred to Psychiatric clinic and Golestan Hospital, in Ahvaz, Iran over 6 months in 2011. An informed consent was obtained from each patient. Patients diagnosed with mild or moderate MDD for six weeks, between 18-55 years of age were included.21 Optioned Beck Inventory Depression was performed before treatment and only the patients who earned 16-46 points were taken in to account in the study. Beck Depression Inventory is a test to diagnose major depression and is used to measure depression severity. This is a questionnaire filled out by subjects including 21 items that each item is scored 3-0. For assessment Dr. Kaviani’s standardized questionnaire adapted for the Iranians, was used in which the score 0-15 meant no sign of depression, 16-31 meant mild depression, 31-46 meant moderate depression, and 47-63 was classified as severe depression (21). Diagnoses were done by psychiatrist.

Patients with the following criteria were excluded from this study: pregnancy and lactation, the presence of clinically significant organic or neurological disorders, Axis II disorder, comorbid disorder in Axis I, consumption of alcohol and other addictive substances except nicotine and caffeine, symptoms that caused or worsen psychotic depression symptoms, symptoms that required hospitalization and emergency action, patients with suicidal thoughts, history of receiving electro convulsive therapy in the last three months, any allergies to medicine particularly Hypericum perforatom , taking lithium, anticonvulsants, sumatriptan, L. dopa, SSRI, buspirone, ergot compounds, selegiline, stimulants, anti-congestive medications, contraceptives, cimetidine , theophylline, thyroid hormones. At the beginning of the study, blood tests were done for blood sugar, fat and the liver and kidney function; patients were excluded if test results were not normal.

Patients were randomly distributed in 4 blocks with 10 patients in each. 5 out of 10 patients took medicine (group I) and others received placebo (group II). Following medications were used: group I took a moderate dosage of tricyclic antidepressants (nortriptyline 75-100 mg daily, imipramine & amitriptyline 100-150 mg daily ) and perforan pills (providing 300μg total hypericin) three times a day, group II took the same dosage of tricyclic antidepressants as the first group did, and placebo pills three times a day for 6 weeks. Their depression was evaluated with Beck Depression Inventory at weeks 0, 3 and 6.

### 3.1. Statistical Analysis

SPSS software was employed to analyze the data. Data were provided by the MEAN ± SEM. P values 0.05 were considered as significant. One-way repeated measure ANOVA was used to compare mean changes. The percentage was used for qualitative variables. Demographic data and statistical analysis were performed using ANOVA-chi-Square and Fisher’s exact tests.

## 4. Results

Data analysis indicated that the mean age of placebo group was 30±16.6 and the mean age of perforan group was 29.8±6.2. Disease duration in perforan group was 30.5 ± 10.56 weeks and in placebo group was 33.81 ± 11.1. No significant differences in age and disease durations were seen between the two groups. In the placebo group there were 11 females and 9 males, and in the perforan group, 10 females and 10 males, no significant difference between the two treatment groups were noticed.

Table 1 shows the mean scores of depression in patients treated by perforan, in the zero week it was 36 and in the third week 28 and at the end of the treatment period 22. According to Table 2 mean differences in depression scores in the beginning of the treatment and the third week was 9. Obtained T value was 1.78 which, considering the statistical level of 0 .08was not significant. The interesting note was that the observed differences in the recovery rate were close to significant. According to Table 2, mean difference in depression scores in the third week of the treatment period and the sixth was 5. The obtained T value was 2.68 which wasnot significant at the statistical level of 0 .3. In the placebo group, according to Table 1 recovery at the zero and the third week of treatment were compared and the mean depression scores were 5. The obtained T value was 2.82 which was not significant at the statistical level of 0.7. According to Table 2, the recovery rate in the third week was compared to the sixth week, the mean difference in depression scores was 1. T value was 8.22 so it was not significant at the statistical level of 0.9.

According to Table 3, the sixth week of the recovery in the two treatment groups were compared. The mean difference in depression scores was 6. The obtained T value was 1.34 so the statistical level of 0.04 was significant. This means that by adding perforan to treatment, compared with placebo, mild to moderate depression can be improved significantly. In this study, side effects, as each patient reported, were recorded and at the end of the study were compared. The quality of sleep significantly increased in the group treated with perforan. Gastrointestinal complications in patients who were treated with perforan were significantly lower than those of the placebo group. In patients who were taking perforan, increase of energy and reduction of fatigue and impatience was reported. . In perforan group three female patients developed mild photosensitivity, the drug was continued and using sun screens improved the situation.

**Table 1:** Mean, Standard Deviation, Minimum Score, Maximum Score and the Number of Patients of the two Groups

Variable group	Mean Beck score	Sd	Min	Max	Number
Beck score zero week(perforan)	36	44.0	0	47	20
Beck score third week (perforan)	27	38.4	0	32	20
Beck score sixth week (perforan)	22	96.4	0	24	20
Beck score zero week (plasebo)	35	37.0	0	47	20
Beck score third week (plasebo)	30	58.7	0	36	20
Beck score sixth week (plasebo)	29	96.7	0	34	20

**Table 2:** Comparison of Depressed Patients Improvement of the Two Groups

Variable Group	Mean Difference	T value	Degrees of Freedom	Significant Level
Beck score in zero week to third(perforan)	9	1.78	29	0.08
Beck score in third week to sixth(perforan)	5	68.2	29	3.0
Beck score in zero week to third(placebo)	5	82.2	29	7.0
Beck score in third week to sixth(placebo)	1	22.8	29	9.0

**Table 3:** Comparison of Recovery Rates of Depression in Patients with Perforan Added to the Treatment of Patient who Placebo was added to their Treatment

Variable	Difference Mean	T value	Degree of Freedom	Significant Level
Beck score of two group in sixth week	6	1.34	56	0.04

## 5. Discussion

In many studies, Hypericum perforatom has proved to have therapeutic effects on depression. In some studies, the effect has been equal to placebo and in some of them it had significant effect. In the current study, the effect of the medicine versus placebo was significant. Considering that the conditions of preparation can alter plant compounds and the studies conducted in different regions used different quantities and were carried out with different vegetable combinations (11), the heterogeneous results can be justified. Adding a tricyclic antidepressantin a moderate level could increase perforan efficacy in this study.

Inthe current study, Beck Depression Inventory, indicated differences close to significant in patients of the perforan group at the end of the third week compared to the zero week. The change was not statistically significant in the sixth week compared to the third week. Perhaps such a result, presumed that if perforan is used as the antidepressant medication, we expect to see the most efficacy in the first three weeks of treatment. This finding had not been reported in the previous studies. This inference was perhaps due to small sample size or study period or was related to the study design. In the current study, the manufacturer`s recommended dose was considered.

A change in the dosage of medications could alter the results. For example, increasing the medicine can lead to better results. As is often, one of the best ways to get antidepressant treatment response is increasing the medication dosage. Placebo group patients reported more mild gastrointestinal problems than the perforan group. May be that’s because of the use of calcium carbonate in the placebo and perforan’s effects on the serotonergic system. None of these cases led to discontinuation and symptoms were improved by recommending medicine with meals. In previous studies discontinuation due to side effects was unusual as well, however, in some of the previous studies gastrointestinal complications were the most common complication of Hypericum perforatom.

Another side effect was photo sensitivity in previous studies, sometimes was referred to as the most common complication. This applied to the current study as well but its rate was very low and given the very high number of sunny days in the place of study, it was perhaps due to previous use of sunscreen, patient’s lack of leaving indoors due to depression, or the difference in plant compounds. Despite the likely reaction to medications that affect serotonin, in the current study no problems of autonomic nervous system instability were reported, maybe because the tricyclic drug was administered in moderate dosage quantities. None of the patients in both groups had complained about sexual side effects which was consistent with other studies and it was an advantage of using this herbal medicine.

Sleep quality improved more in perforan group and the energy increased. This improvement had not been reported in previous studies. In previous studies, drowsiness and fatigue were listed as medical side effects which were not present in the current study. Regarding the potential effects of this medicine on GABA receptors, the effect was probably due to different materials in the Iranian sample. These findings favor the superiority of this medication on serotonin reuptake inhibitors in the field of sleep. Sleep problems were common complications of specific serotonin reuptake inhibitors.

### 5.1. Suggestions

It was the first study on this subject in Iran. Another advantage was to study the use of placebo. In many of the patients, depression is a chronic disease and requires long term treatment. In order to study the long term effects of this medication, it is suggested that further studies be done over a longer period of time.

The proposal would be to design another study in which Beck Depression Inventory is divided into several subgroups and perforan effect be assessed in each category and period of effectiveness separately. Giving patients the tendency to use herbal medicine, the use of this medication can be considered in the treatment of other psychiatric disorders such as anxiety, post-traumatic stress disorders, and sleep problems, according to the possible unknown mechanisms of perforan. Perforan was added to tricyclic antidepressants; further studies could be done to compare perforan with other antidepressants to find the effects of this medication.

### 5.2. Limitation

Limitations of this study were the small group of patients and the short study period. Because of legal restrictions perforan could not be used as a single treatment. The absence of a complete inventory of the adverse effects and the lack of possibility to follow up participants were other limitations of this study.
